# Non-Genomic Effects of Xenoestrogen Mixtures

**DOI:** 10.3390/ijerph9082694

**Published:** 2012-07-31

**Authors:** René Viñas, Yow-Jiun Jeng, Cheryl S. Watson

**Affiliations:** Department of Biochemistry and Molecular Biology, University of Texas Medical Branch, Galveston, TX 77555, USA; Email: revinas@utmb.edu (R.V.); yjeng@utmb.edu (Y.-J.J.)

**Keywords:** non-genomic, estrogenic mixtures, endocrine-disrupting chemicals, xenoestrogens, non-monotonic dose-response curves, kinases, hormesis

## Abstract

Xenoestrogens (XEs) are chemicals derived from a variety of natural and anthropogenic sources that can interfere with endogenous estrogens by either mimicking or blocking their responses via non-genomic and/or genomic signaling mechanisms. Disruption of estrogens’ actions through the less-studied non-genomic pathway can alter such functional end points as cell proliferation, peptide hormone release, catecholamine transport, and apoptosis, among others. Studies of potentially adverse effects due to mixtures and to low doses of endocrine-disrupting chemicals have recently become more feasible, though few so far have included actions via the non-genomic pathway. Physiologic estrogens and XEs evoke non-monotonic dose responses, with different compounds having different patterns of actions dependent on concentration and time, making mixture assessments all the more challenging. In order to understand the spectrum of toxicities and their mechanisms, future work should focus on carefully studying individual and mixture components across a range of concentrations and cellular pathways in a variety of tissue types.

## 1. Introduction

Trace levels of industrial and naturally occurring chemicals have been shown to perturb endocrine systems. These endocrine disrupting chemicals (EDCs) are currently the subject of intense research and regulatory action. A large number of these EDCs act via the estrogen receptor (ER), imperfectly mimicking and interfering with the physiologic actions of endogenous estrogens. Xenoestrogens (XEs) can bind to ERs in the cell nucleus, where the complex recognizes DNA response elements and alters gene expression; in the non-genomic pathway XEs can bind to membrane-bound ERs and rapidly initiate signaling cascades that culminate in kinase and phosphatase activations, ultimately influencing cellular function by post-translational modifications of a variety of proteins [1]. 

Functional consequences observed at the organismal level include decreased fecundity in aquatic organisms, altered sexual behavior and memory in rats, and malformations and decreased mobility of human sperm [2,3,4,5,6,7]. XEs have also been implicated in the development of such chronic diseases as obesity, diabetes mellitus, asthma, and cancer [8,9,10,11,12]. Many XEs, such as nonylphenol, are persistent organic byproducts of our industrialized society that can remain in the environment for extended periods of time, thereby increasing the likelihood of human and wildlife exposure [13,14]. Human exposure to XEs can result from eating or drinking contaminated food and water that has been kept in plastic containers constructed from XEs such as bisphenol-A (BPA) [15]. Waste water and subsequently drinking water have also become plentiful sources of XEs (e.g., pharmaceuticals, surfactants) that are not removed by treatment plants [16,17]; in addition, use of chlorine as a typical means of water purification has given rise to poly-chlorinated conjugated compounds that can also interfere with endocrine regulation [18]. 

In real-world situations, however, humans and wildlife are not exposed to one chemical at a time, but rather to complex mixtures. The potential health hazard from EDC mixtures is one of the most difficult challenges for a regulatory agency to evaluate. Nevertheless, several legislative mandates (Superfund Amendments and Reauthorization Act (1986); Food Quality and Protection Act (1980); Safe Drinking Water Act (1996)) require the U.S. Environmental Protection Agency to examine mixtures of chemicals in regulatory decision making [19]. Studies examining known XEs in combination with endogenous estrogens (binary mixtures) have shown additive, synergistic, or antagonistic changes in cell signaling (mitogen-activated kinases (MAPK)) and functional (proliferation and peptide hormone (prolactin, PRL) release) endpoints [20,21,22,23,24,25,26], demonstrating the difficulty of predicting the estrogenic responses in XE mixtures. There is also strong evidence that several types of XE mixtures can produce non-monotonic dose-responses at low concentrations, making the task of risk assessment all the more difficult [27]. Others have recently reviewed the literature on the genomic responses contributing to endocrine disruption, and we refer readers to those reviews [27,28,29]. In this review we will focus on the non-genomic actions of diverse estrogenic chemicals, as single entities and as mixtures, as well as current approaches used to evaluate their toxicity levels.

## 2. Mechanisms of Estrogenic Actions-Genomic & Non—Genomic Pathways

### 2.1. Different Types of Estrogen Receptors

Estrogens, other steroids, and related compounds were traditionally thought to mediate their actions exclusively via nuclear actions, so the name “nuclear receptor” has become exchangeable with “steroid receptor.” ERs are one category of nuclear receptors in the larger family through which EDCs can act. Others are the receptors for compounds such as androgens, thyroid hormones, aryl hydrocarbons, and pregnane-x compounds [30,31,32,33,34]. Nuclear actions result in various macromolecular syntheses initiated by transcription factors, and require extended time periods to elicit a response. Genes regulated by estrogens via this pathway influence reproduction, development, bone integrity, cardiovascular function, behavior [35], and a growing list of tissue-specific functions. However, compelling more recent evidence has resulted in wide acceptance of an alternative non-genomic, often rapid, signaling pathway for estrogenic actions [36,37,38,39]. Membrane-bound ER subtypes (α, β) have been identified and linked to the initiation of non-genomic responses [36,37,40]. Even more recently, another entirely different receptor type has been associated with rapid estrogenic responses—a seven-transmembrane G protein-coupled receptor called GPR30 (also referred to as GPER) [41,42]. 

The reason for the existence of different ER localizations and subtypes is not yet clear. We have yet to document enough examples of the serpentine-type GPR30 receptor actions to determine if it fits discernible subcategories of functional responses. Different ERs (membrane and nuclear) could exist to accommodate the wide variety of estrogenic molecules with distinct functions [43], as mERs in lipid membrane environments as opposed to nuclear aqueous environments might take on different conformations and thus accept different subsets of ligands. Unfortunately, this ability of ERs to accommodate many different lipophilic compounds also makes them vulnerable to EDC binding. It is also possible that multiple ERs must all participate for a complete and complex cellular response [44]. 

Membrane-initiated signaling may be a “first alert system” for the cell to eventually prepare for more permanent make-overs [43]. Activation of MAPKs and other kinases do in some cases lead to activation of transcriptional events [22], resulting in new proteins and altered cellular differentiation. However, some non-genomic responses end in complete functional endpoints without the eventual induction of genomic endpoints. For example, we have previously shown that in pituitary cells, mER-initiated pathways are capable of activating caspases 8 and 9, as well as inducing ion influxes triggering the release of PRL from secretory vesicles [22,26]. 

Our group and others have explored the similarities between the mERα and the intracellular estrogen receptor (iERα) [45,46]; a close similarity was established between alternatively localized forms when nine iERα-specific antibodies (Abs) recognized seven different mERα epitopes in unpermeabilized cells where Abs cannot cross the plasma membrane [44,45]. Additionally, the ability of the ERα-recognizing Ab H151 to elicit responses or block subsequent responses to estrogenic ligands also added strength to this identification [44,47]. 

Other results have also suggested identity of ERα in both subcellular locations by virtue of the proteins with which they associate. Recently, we used Duolink immunofluorescence imaging to visualize the partial co-localization of mERα and the G_αi_ subclass of G proteins at the cell membrane. Interactions of ERα and caveolin-I were also demonstrated by epitope proximity ligation studies which supported the idea that these proteins jointly participate in estrogen-induced signaling in the membrane [48,49,50,51].

The mERα was also deemed identical to its nuclear counterpart in MCF7 breast cancer cells by membrane isolation (affinity chromatography) and mass spectrometry analysis [52]. These results are in line with the blocking of responses by antisense [53] and siRNA knockdowns [52] of ERα, and the use of a variety of immunohistochemistry techniques for mER identification [53,54]. More recently, the mechanism for membrane attachment has been shown to be via post-translational palmitoylation [55]. Thus overall, it is very likely that mERα is indeed closely related to iERα, modified for targeting to the plasma membrane.

### 2.2. Types of Non-Genomic Signaling Induced by Estrogens and Xenoestrogens and Their Functional Consequences

BPA and other XEs have been found to be “weak” inducers of estrogenic activity via the genomic pathway in comparison to E_2_ (1,000-fold difference) [56]. However, BPA is equipotent with E_2_ in its ability to initiate rapid non-genomic responses from membrane receptors [57]. Non-genomic signaling can occur within seconds-minutes of the initial steroid-receptor contact, yet sustained activation of cell signaling can influence more permanent changes such as cell proliferation, differentiation, movement, or apoptosis. Membrane steroid receptor-mediated signals include the activation of kinases that regulate the phosphorylated states of important functional proteins, each linked to different pathways of actions. In our studies, we have extensively examined the estrogen- and XE-induced MAPK signaling activations, specifically those of the extracellular regulated kinases (ERKs 1 and 2), the c-Jun N-terminal kinases (JNKs 1 and 2), and the p38 kinase. Activation of ERKs is commonly associated with cell growth and survival, whereas activation of JNKs has long been linked to the induction of apoptosis [58,59,60,61]. In the case of E_2_, coumesterol, and BPA, we have been able to correlate strong and sustained ERK activity together with weak JNK activation responses to the induction of cell proliferation in the GH3/B6/F10 pituitary cell line [22,26]—the ERK response apparently predominating. Others have speculated that extended stimulation of the JNK pathway may lead to a shutdown of ERK and its associated effects [59]. Therefore, there is interplay between these integrator kinases to render a final outcome.

Ion fluxes (Ca^2+^, K^+^. Na^+^, H^+^) are a common signaling responses to steroids and related compounds, often leading to changes in cell mobility, downstream signaling processes, and peptide hormone secretion [43]. Using an mERα-enriched GH3/B6/F10 prolactinoma cell line we examined functional consequences of elevated Ca^2+^ levels (PRL release) upon stimulation with low picomolar and sub-picomolar E_2_ concentrations [22,26,37,57,62,63]. BPA, o′,p′-dichlorodiphenyl-ethylene (DDE), nonylphenol, coumesterol and other known XEs caused release of PRL from secretory vesicles within a minute, with non-monotonic dose response characteristics. Changes in Ca^2+^ influx were monitored in order to establish a correlation between Ca^2+^ levels and PRL secretion [26,57,63]. Interestingly, Ca^2+^ fluxes were not non-monotonic, and thus did not explain the bi-modal nature of the changes seen with PRL release. Therefore, for this and other reasons, it is likely that regulation of peptide secretion also involves additional signaling pathways [26,43,57]. 

We have found that non-genomic effects of estrogens also modulate transporter functions [20,21,64]. Using the rat pheochromocytoma (PC12) cell model, we examined dopamine efflux via the dopamine transporter upon exposure to several physiologic estrogens at 10^−14^–10^−8^ M concentrations. Our studies found that like amphetamines, multiple estrogens [65] are capable of reversing transport direction of the dopamine transporter via kinase regulation [21,66]. Upon XE exposure, all compounds tested (nonylphenol, BPA, dieldrin, endosulfan and DDE) elicited dopamine efflux with non-monotonic response characteristics, resembling a U-shaped curve, or with even more fluctuations in the responses, thus making it difficult to extrapolate low dose effects from high ones to assign chemical safety margins for regulatory purposes. 

### 2.3. Non-Monotonic Dose Responses of Xenoestrogens

According to the *International Dose-Response Society* (http://www.dose-response.org), hormesis is defined as “a dose-response phenomenon characterized by low-dose stimulation and high-dose inhibition”[67]. The occurrence of such non-monotonic responses to XEs at low concentrations (below the so-called toxic threshold) has in recent years gained increasing awareness by the scientific and regulatory community [68]. Still, there is considerable debate as to the fundamental mechanisms responsible and their practical use in evaluating chemical safety. Typical dose-response studies in regulatory testing involve *in-vivo* or *in-vitro* models exposed to high concentrations of chemicals uncommonly found in human populations or the environments to which they are exposed [69,70]. Past evaluations assumed that all chemical responses follow a linear monotonic path that eventually reaches an asymptote; safe doses for humans or wildlife were then determined to be just below the lowest measurable response-causing concentrations or the no-observed-effect-level (NOEL) [27]. However, most XE exposures occur at low doses and exhibit non-monotonic responses that make it difficult to predict low-dose effects from high-dose effects [27]. Furthermore, because XEs are rarely present at concentrations that produce immediate death or illness, traditional toxicology testing is irrelevant, and in any case insufficient for understanding XE mechanisms [27,71,72,73]. Therefore, more recent XE studies have begun to investigate low dose exposures focusing on very sensitive endpoints such as cell signaling or gene expression that could have dire repercussions on tissue and whole-animal functioning and health over time [74]. 

Various theories have been offered as explanations for non-monotonic dose responses; these have been previously reviewed [27,73,75,76,77,78,79] and so will only be summarized here. We and others observed that XEs are capable of initiating multiple receptor-proximal signaling cascades, responding with different rates and dose dependencies; these eventually contribute to composite response patterns of downstream phospho-activated MAPKs (*i.e.*, *p*ERK, *p*JNK, *p*38) [80,81,82,83]. It is well known that inhibition or negative feedback regulation of MAPKs is crucial for preventing unfavorable effects from extended pathway stimulation [58]; hence, as seen in many of our studies, when concentrations of both physiologic estrogens and XEs increase (10^−15^–10^−7^ M), MAPK responses eventually decrease [22,23,24,25,26,84]. Furthermore, assessment of resulting functional endpoints has also shown that low doses and short exposure periods induce responses (e.g., proliferation and PRL secretion in pituitary cells), while higher doses and longer exposure periods cause inhibition [22,27,57,75,85,86]. Other plausible explanations for non-monotonic dose-responses as a means of preventing overstimulation from XEs at higher concentrations include receptor down-regulation or desensitization, changes in receptor selectivity when going from low (selective ER binding) concentrations to high (non-selective) concentrations, the presence of co-factors or co-regulators that influence hormone-receptor binding at certain selective concentrations, and the presence of multiple receptor subtypes that bind to the same XE, but each with a different (stimulatory or inhibitory) response pattern [27,73,87,88].

For mixtures toxicology, the significance of non-monotonicity has not been adequately characterized [89]. This is partly due to the impossibility of testing so many chemical interactions in mixtures where components can target various mechanisms and vary by tissue. In addition, chemical interactions such as synergy and potentiation occur in the low dose stimulatory zone, below the traditionally identified toxicological threshold [89]. Furthermore, such responses are probably limited by various biological constraints, to modest increases of 30–60% above controls [89,90]. In addition, evidence exists for EDCs inducing biological effects even at very low analytically undetectable concentrations. “No-threshold” responses [27,91,92] can be due to the presence of endogenous or exogenous mimetic hormones already present. These obscure low dose responses of compounds being experimentally tested [27,91,92], unless effectively removed (such as in well-controlled cell culture experiments).

## 3. Types of Estrogens and Estrogen Mimetics

### 3.1. Physiologic Estrogens

Produced primarily in the testes and ovaries, estrogens such as estradiol (E_2_), estrone (E_1_), and estriol (E_3_) play diverse roles in human and wildlife physiology beyond those required just for reproductive success, affecting metabolism, bone integrity, cardiovascular functions, behavior and mood, and other functions [93,94]. Physiologic estrogens play selective roles in women’s life stages. For example, the predominant hormone driving sexual development, function of reproductive organs (e.g., breast and uterus) and the menstrual cycle is E_2_. E_1_ is found at elevated levels (~150–200 pM) during post-menopausal stages, while E_3_ is high during pregnancy (~10–100 nM); males also have lower development stage-specific blood levels of endogenous estrogens [95]. Excessive estrogenic activities have been associated with the development of cancer in estrogen-responsive tissues (e.g., breast and uterus). Decreased levels of E_3_ have been linked to complications of eclampsia and an increased probability of Down’s syndrome in offspring [96,97]. Therefore, understanding the mechanisms that influence or disrupt all estrogenic actions is crucial for preventing negative outcomes. Compared to E_2_, these other endogenous estrogens have yielded weak genomic responses [98,99]. However, we and others have shown that many of these same compounds can potently activate non-genomic signaling pathways [21,24,25,100,101,102], which means that they can affect health and life stage-selective functions.

### 3.2. Pharmaceutical and Personal Care Product Estrogens

Pharmaceuticals and personal care products enter urban sewage networks and wastewater treatment plants, and eventually streams and waterways, via human use. Pharmaceuticals are excreted after use and therefore enter sewage from various locations, but especially from hospitals [103]. Once in aquatic environments, low molecular weight and hydrophilic compounds will dissolve in water or will degrade in the sewage sludge [104]. Still, a large amount of pharmaceuticals and their metabolites will remain, and expose humans via drinking/bathing water or by consuming fish and other aquatic animals that have ingested or absorbed pharmaceutical residues. Examples of estrogenic compounds commonly found in aquatic systems are ethinyl estradiol and mestranol (commonly used as hormonal contraceptives), trenbolone (used for growth promotion in cattle), tamoxifen (and other anti-estrogens used for breast cancer treatment that can sometimes be estrogenic in certain tissues), and equine or other estrogens used for postmenopausal hormone replacement, many of which are ubiquitously present in our water supplies in the ng/L, ppb, nM range [104,105,106,107,108,109]. Their estrogenic effects via the non-genomic pathway, as single chemicals and as mixtures, are currently unexplored.

### 3.3. Phytoestrogens

Some plant-derived components of the diet can act as either estrogenic agonists or antagonists of mERs, depending on concentration and tissue specificity [22]. Common sources of phytoestrogens include soy-based products such as tofu (isoflavones and their metabolites); sprouts, red clover, or alfalfa (coumestans); and flaxseed, sesame seed, or nut products (lignans). In Asian cultures traditional culinary dishes are rich in phytoestrogens that are a major component of dietary intake. Better bone health, lower cardiovascular and cancer risks, and extended lifespans are often attributed to phytoestrogens in Asian diets. The intake of soy can be as high as 50 g a day, with measured genistein plasma concentrations from 0.1–10 µM [110,111]. In contrast, Western diets typically have ten-fold lower concentrations [112]. Another phytoestrogen, the stilbenoid resveratrol, is plentiful in red wine and other grape products, and has enjoyed much attention for its potential anti-diabetic, anti-cardiovascular disease, and cancer prevention effects, especially in cultures with a rich wine heritage [113,114]. 

An ever-expanding number of studies explore the physiologic and biochemical outcomes of phytoestrogen use. Such studies have been prompted by widespread phytoestrogen use as replacements for estrogen loss at menopause (hormone replacement therapy); phytoestrogens may prevent the increased risk of cancer that can occur from taking other estrogenic hormone supplements [115,116,117]. In a recent study we determined that unlike the pharmaceutical estrogen diethylstilbestrol, high levels of phytoestrogens do not promote precancerous growth of the pituitary and other estrogen-responsive tissues in Fischer 344 rats [118]. Though high concentrations of phytoestrogens may not cause adverse effects in an adult individual, for an infant, the effects could lead to adverse developmental repercussions [119,120]. Serum levels of genistein have been detected in a range of 1–10 µM in infants exclusively fed soy-based formulas [121,122]. Phytoestrogens have been reported to have low transcriptional activity via yeast-based receptor assays [123], but they are capable of inducing MAPK signaling via the membrane ER (mERα) at doses far below or equivalent to the reported plasma concentrations achieved with even Western diets [22].

Unless taken individually as dietary supplements, phytoestrogens are typically found in the diet as chemical mixtures. Therefore the health benefits of resveratrol, for example, could be the result of an additive effect with one of the other hundreds of “minor phenols” found in red wine [124,125], and also the grape species type that affects the wine’s chemical composition. Our lab has shown that in a prolactinoma cell line model, resveratrol attenuates cell proliferation when found in combination with E_2_ [22]. We therefore must examine mixtures of phytoestrogens and their combinations with endogenous estrogens more carefully for their beneficial (and deleterious) effects, as well as the cross-talk between genomic and non-genomic pathways. Much research remains to be done to even begin to understand how phytoestrogen mixtures may combine the signaling effects of their component compounds.

### 3.4. Synthetic/Anthropogenic Estrogens

Anthropogenic or man-made chemicals with estrogenic capabilities have become abundant pollutants in our environment (air, soil and water); many are by-products of plastics, preservatives, industrial surfactants, and pesticides. Agricultural compounds (e.g., pesticides such as dieldrin and endosulfan) have been detected in breast milk, urine, maternal blood, and serum in appreciable amounts, with links to low birth weight, fetal death, and childhood cancers [126,127,128]. Alkylphenols are surfactant breakdown products that are have been shown to be highly estrogenic via non-genomic and some genomic pathways [25,26,129,130]. After the recent (2010) Deepwater Horizon oil spill in the Gulf of Mexico, large amounts (2,900,000 L) of oil dispersants containing alkylphenols were used, increasing safety concerns over their estrogenic effects [131]. In particular, nonylphenol can remain in the environment long enough to bio-accumulate in humans and wildlife (including human food supplies), leading to developmental abnormalities [132].

The highly controversial XE, BPA, has also been detected in significant levels (in urine samples) in 93% of U.S. residents ≥ 6 years of age. BPA’s prevalence is due to its incorporation into many manufactured goods that we as consumers use on a daily basis; these include: plastic food and water containers, thermal coatings on cashier paper receipts, linings in canned foods, and dental repair materials, among many others [133,134,135,136,137]. Exposure to BPA during critical developmental stages has been implicated in the onset of a variety of health problems, including breast and prostate cancer, asthma, diabetes and reproductive dysfunction [9,10,138,139]. Growing concern over BPA has prompted 11 U.S. states, Canada (2008), and Europe (2011) to ban its use in plastic feeding bottles for infants [140,141,142,143]. Though much information has been amassed on BPA’s estrogenic actions, only recently have we and others explored its effects on functional endpoints at the cellular level (peptide hormone release and cell proliferation) via non-genomic mechanisms [21,22,23,25,144,145,146,23,25,144] as a potential mechanistic explanation for EDC-induced disease. 

Pesticides/herbicides are perhaps the most studied of this class of chemicals in mixtures, as multiple remedies are often applied against different insect and weed categories simultaneously for efficiency [29,147], and increased regulatory constraints have demanded more scrutiny [148]. However, very few studies have evaluated the low-dose non-genomic estrogenic effects of this class of compounds when administered either alone or in mixtures [149]. With increased awareness of the need to study chemical mixtures’ actions via more recently revealed estrogenic signaling mechanisms, an increasing number of studies have emerged, which we will discuss in the following section.

## 4. Non-Genomic Actions of Estrogen/Xenoestrogen Mixtures

Various approaches for evaluating chemical mixtures have been proposed by the scientific community [148]; however, there is no internationally agreed-upon procedure. The proposed approaches fall within two general categories, the whole-mixture (evaluation as though mixtures are single entities) and the component-based (evaluating individual chemicals in a mixture to estimate response) approaches [72,150,151,152]. Whole-mixture approaches can be impractical due to the multiple interactions that can potentially occur in real-world mixtures, some of which do not necessarily occur via a common mode of action by structurally similar compounds. Furthermore, this approach does not identify which types of chemical interactions are responsible for additive, synergistic, or antagonistic effects. Most studies have thus resorted to using the component-based approach, which requires information on each individual component within the mixture [150]. The component-based approach operates on the calculated sum from either of the following methods: (1) *concentration or dose addition method*, which assumes that mixture components act on a similar target and therefore elicit a common response; and (2) the *response addition method*, which assumes that components act on different targets (the overall response is calculated from individual components) [148,152]. The latter method is not commonly used for XE mixtures. 

The overall goal of every method is to establish principles of how chemicals behave based on structure and mode of action. Once enough examples are processed and the model optimized, then predictions for unknown mixtures should be possible. In a review, Kortenkamp extensively discussed mixture effects of several classes of EDCs (*i.e.*, estrogenic, anti-androgenic, and thyroid-disrupting agents) [28], focusing primarily on their genomic and functional responses. Below, we will briefly focus on the few existing non-genomic studies of synthesized estrogen mixtures.

Jeng and Watson studied the phospho-activation of MAPK (*p*ERK) upon exposure to binary mixtures of endogenous estrogens (E_1_, E_2_, and E_3_) at single physiologic (nM) concentrations with increasing (10^−15^–10^−7^ M) concentrations of alkylphenol compounds in the GH3/B6/F10 pituitary cell line [25]. Individual compounds caused non-monotonic dose-responses, but with varying weak, moderate or strong response levels compared to E_2_. The composite responses were not additive, and often showed attenuation at the higher concentrations. The degree of attenuation was based on the response magnitude and potency of the paired xenoestrogen. The stronger the XE’s activating response, the more it was able to attenuate the physiologic estrogenic response. 

When assessing the effects of XEs on dopamine efflux through its transporter in PC12 cells, Alyea and Watson also found that 10^−14^–10^−8^ M DDE caused a weak efflux as a lone compound, but in a binary mixture with 10^−9^ M E_2_ it additively enhanced dopamine efflux. BPA in contrast evoked a strong efflux response on its own, but when mixed with 10^−9^ M E_2_ it inhibited efflux [64].

**Figure 1:**
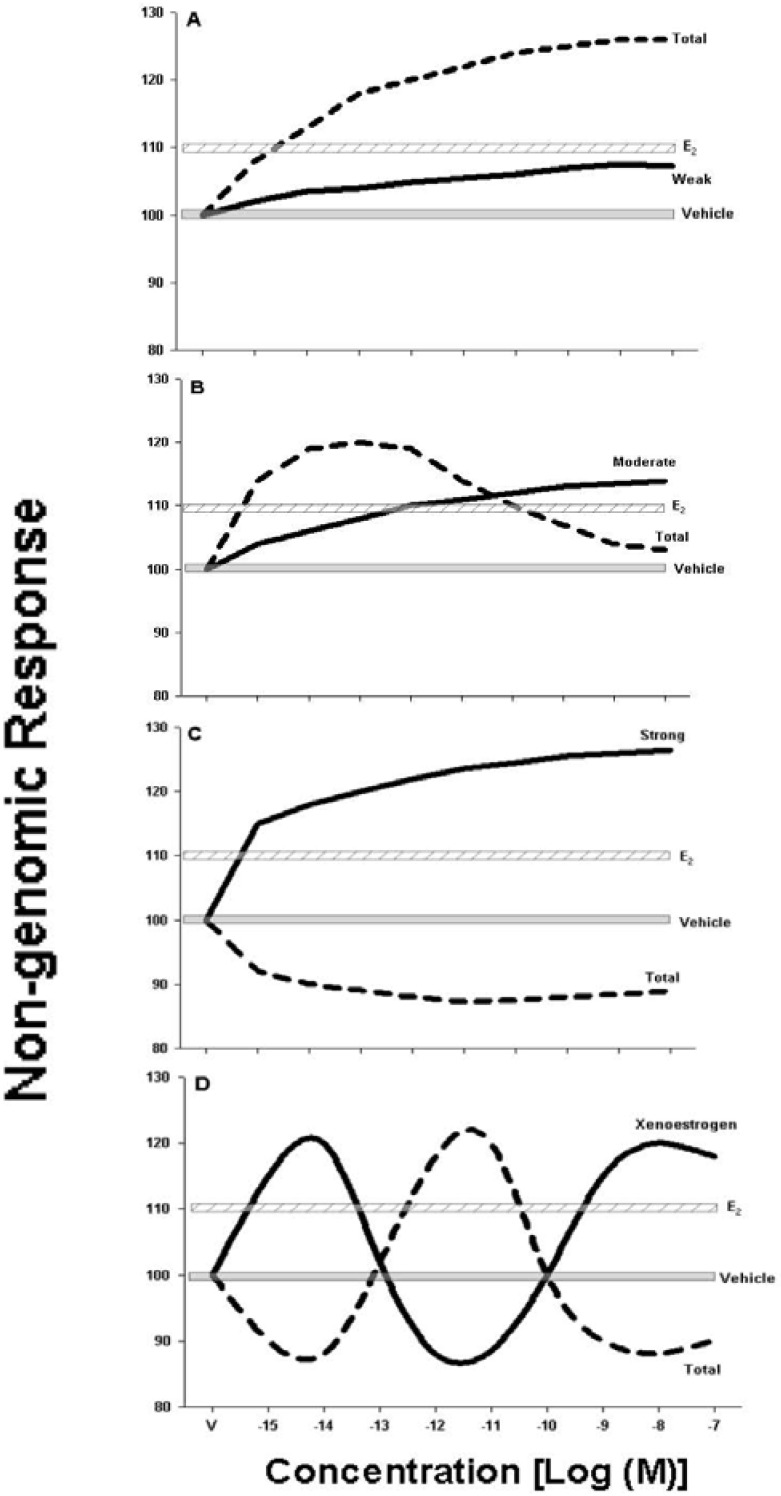
Working model of xenoestrogen (XE) alteration of physiologic estrogen non-genomic response effects. XEs of increasing dose were used to challenge the responses of the physiologic estrogen estradiol (10^−9^ M E_2_). These combinations examples are mainly summarized from [25,26]. In each case the vehicle control (V) and 10^−9^ M E_2_ responses are shown by horizontal bars. The response to an XE alone is shown by a solid line, and the combination of 10^−9^ M E_2_ plus the XE is shown by a dashed line. The types of combination responses are: (**A**) A weak XE enhances the physiologic estrogen E_2_response; (**B**) A moderate XE response enhances the E_2_ response at low concentrations, and inhibits it at higher concentrations; (**C**) The strongest XE inhibits the E_2_ response at all concentrations, with increasing inhibition as the XE concentration increases; and (**D**) If the XE exhibits a fluctuating non-monotonic estrogenic response, the effect on the E_2_ response also fluctuates, in line with cases A–C above. These idealized data summarize what we have seen in combinations using a variety of XEs. Depending on the estrogenic potency of the XE, when paired with a physiologic estrogen, an inverse relationship in responses occurs (enhancement or attenuation).

The overall pattern observed in these two studies was that when a compound with a weak response is paired with a physiologic estrogen, the response is enhanced. But, when a compound elicits a potent estrogenic effect, then it inhibits the paired physiologic estrogen’s response. This progression is summarized graphically in Figure 1. In a very recent tertiary mixture study, we have observed further inhibition of responses to the physiologic estrogen E_2_ by two added XEs However, the same mixture resulted in a synergistic positive response for *p*JNK (Viñas and Watson, unpublished); hence, when assessing non-genomic pathways one has to take into consideration the variety of signaling responses, and probably the interactive nature of signaling “webs”. Response inhibitions by combinations of estrogens may be governed by cellular protective mechanisms against combined hormone overstimulation. Overstimulation can be wasteful and even dangerous when the enhanced function (such as peptide release or cell proliferation, for example) can lead to diseases like cancer.

Interestingly, not all signaling pathways culminating in different functional responses may behave in the same fashion. When another type of response (PRL release) was monitored under the same (binary mixture) circumstances, BPA’s strong *p*ERK response when present by itself did not correlate with a strong PRL secretion. However, PRL release did decrease when BPA was paired with either E_2_ or E_1_ (but not the weaker E_3_) in pituitary cells [24]. This means that one has to study sufficient examples of compounds over a wide range of times points and concentrations, assessed for a spectrum of different signals and functional endpoints. It will take an adequately representative set of such data to finally hone our predictive principles. In addition, for some complex responses such as cell proliferation, there will undoubtedly be both genomic and non-genomic contributory components to consider, as well as cross-talk between signaling pathways.

## 5. Conclusions

Assessing enough examples of XE mixtures to establish predictive principles is still largely beyond our current datasets. To do so will undoubtedly require a large training dataset of both genomic and non-genomic cellular responses, including recognition of signaling cross-talk, and resulting functional outcomes. The effects of XEs can vary greatly, depending upon cell, tissue, and organ type. Complications due to the potential non-monotonic nature of responses and variations in the makeup of mixtures will also require systematic evaluation. Until now, the vast majority of XE studies focused solely on a single chemical’s effects without appreciation for the more realistic conditions of chemical mixtures of XEs. Furthermore, while assessing XE effects on humans or wildlife alone or in mixtures, few studies have addressed the impact of XE combinations with endogenous hormones [22,24,25]. Evaluation of such combinations is critical to a fundamental understanding of endocrine disruptions and resulting diseases. Despite several legislative mandates over the years, evaluation of mixtures is still in its infancy, and we are just beginning to learn how to design practical yet sufficiently comprehensive studies. Sophisticated tools such as mathematical modeling will undoubtedly eventually be necessary to provide new insights into how to interpret responses to the multiple chemical combinations that exist in real life, and to enhance the accuracy of our risk assessment predictions.
